# Measurement of the alcohol biomarker phosphatidylethanol (PEth) in dried blood spots and venous blood—importance of inhibition of post-sampling formation from ethanol

**DOI:** 10.1007/s00216-021-03211-z

**Published:** 2021-02-15

**Authors:** Olof Beck, Maria Mellring, Christian Löwbeer, Sabina Seferaj, Anders Helander

**Affiliations:** 1 Department of Clinical Neuroscience, Karolinska Institutet, 171 77 Stockholm, Sweden; 2 SYNLAB Medilab, 183 34 Täby, Sweden; 3 Department of Laboratory Medicine, Karolinska Institutet, 141 86 Stockholm, Sweden; 4 Department of Clinical Pharmacology, Karolinska University Laboratory, 141 86 Stockholm, Sweden

**Keywords:** Alcohol biomarker, DBS, Microsampling, Phosphatidylethanol, Phospholipase D, Inhibition

## Abstract

Phosphatidylethanol (PEth) is a group of phospholipids formed in cell membranes following alcohol consumption by action of the enzyme phospholipase D (PLD). PEth measurement in whole blood samples is established as a specific alcohol biomarker with clinical and forensic applications. However, in blood specimens containing ethanol, formation of PEth may continue after sampling leading to falsely elevated concentrations. This study evaluated the use of dried blood spot (DBS) and microsampling specimens to avoid post-sampling formation of PEth. Filter paper cards and three commercial devices for volumetric microsampling of finger-pricked blood were assessed, using PEth-negative and PEth-positive whole blood fortified with 2 g/L ethanol. PEth (16:0/18:1) was measured by LC–MS/MS. Post-sampling formation of PEth occurred in wet blood and in the volumetric devices, but not filter paper cards, when stored at room temperature for 48 h. Addition of an inhibitor of PLD, sodium metavanadate (NaVO_3_), eliminated post-sampling formation during storage and drying. In conclusion, the present study confirmed previous observations that PEth can be formed in blood samples after collection, if the specimen contains ethanol. The results further demonstrated that post-sampling formation of PEth from ethanol also occurred with commercial devices for volumetric dried blood microsampling. In order for a PEth result not to be questioned, it is recommended to use a PLD inhibitor, whether venous blood is collected in a vacutainer tube or finger-pricked blood is obtained using devices for dried blood microsampling.

**Graphical abstract:**

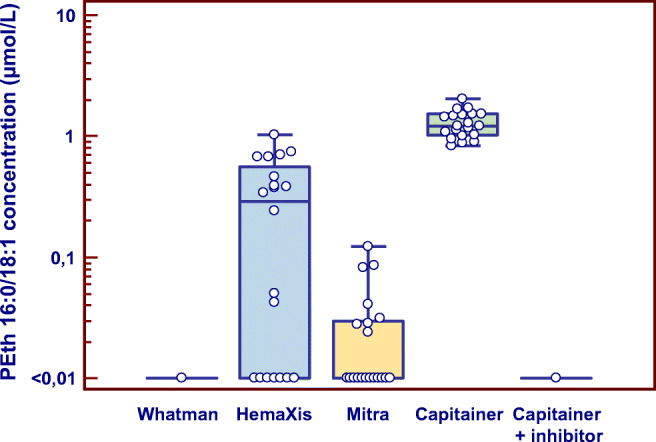

## Introduction

Phosphatidylethanol (PEth) is a group of ethanol-derived phospholipids that are formed in cell membranes in the presence of ethanol [1–3]. In this reaction, which is catalyzed by the enzyme phospholipase D (PLD), ethanol and phosphatidylcholine combine into PEth, comprising of a phospho-ethanol head group, a glycerol backbone, and two fatty acid chains. The two fatty acid moieties can be the same or different, which means there are many possible PEth homologs [4, 5]. The most prevalent ones found in human whole blood after alcohol intake are PEth 16:0/18:1 (i.e., PEth containing one palmitic acid and one oleic acid) and 16:0/18:2 (one palmitic acid and one linoleic acid), which together make up about 60–70% of the total amount [4].

Because formation of PEth requires the presence of ethanol, the PEth concentration in whole blood samples was suggested and introduced as a specific alcohol biomarker [2, 6–8]. Initially, a total PEth fraction was quantified using high-performance liquid chromatography (HPLC) with evaporative light scattering detection [9, 10], but after shifting to selective mass spectrometric detection (LC–MS) [4, 11], PEth measurement has instead focused on the predominant individual homologs. The change to LC–MS-based methods also meant that the analysis became more sensitive, from only being able to detect higher PEth levels occurring after prolonged heavy drinking [3, 12] to also detect lower levels seen after moderate drinking or a single ethanol drinking episode [2, 13–15]. Today, PEth 16:0/18:1 is usually the single target analyte, when PEth is employed as a routine alcohol biomarker with both clinical and forensic applications [11, 16, 17].

Depending on the absence or presence of PEth in a blood specimen, different conclusions are drawn about previous sobriety or, based on the measured concentration, extent, and time of alcohol intake [16, 17], and the result can have important consequences for the person being tested. A confounding factor is that the rates of PEth formation [18, 19] and elimination [20] are both subject to considerable inter-individual variability, which complicates estimation of the amount and time of ethanol intake. Another complication is that PEth formation may continue in the test tube after sampling if the blood contains ethanol, whereas the PEth concentration is stable when specimens are stored at −80 °C [4, 21, 22]. Blood samples for PEth measurement are, however, not always routinely tested for ethanol, and even if ethanol is detected and the specimens are then placed at −80 °C, PEth may already have been formed between the time of sampling and arrival in the laboratory. To eliminate the risk of post-sampling formation, addition of a PLD inhibitor to the blood tubes is a possibility [23]. PEth is otherwise considered to be relatively stable in blood specimens during routine transport and handling [4, 24], not least since erythrocytes lack phosphatidylcholine phospholipase C which catalyzes PEth degradation [3], but the PEth level may decrease in incorrectly stored samples [22, 25].

Collecting capillary finger-pricked blood onto filter paper (dried blood spots, DBS) has emerged as a convenient alternative to venipuncture, by being less invasive and avoiding the need for professional medical staff and special sampling facilities [26]. The use of dried blood microsamples instead of test tubes also simplifies sample transport and storage. Dried blood microsamples have been demonstrated to be useful for the measurement of PEth [25, 27–31], and this was also suggested to eliminate the risk for post-sampling formation, assumed to be linked to the evaporation of ethanol [28], and possibly enzyme inactivation, during drying.

As a follow-up to our previous publication on the usefulness of volumetric dried blood samples for PEth measurement [27], this study was undertaken to investigate the risk for post-sampling formation of PEth (i.e., PEth 16:0/18:1) from ethanol in venous whole blood and in blood samples collected on standard filter paper cards and using three commercial dried blood microsampling devices. The usefulness of PLD inhibitors to prevent PEth formation from ethanol was also examined.

## Experimental

### Chemicals and dried blood microsampling devices

The PLD inhibitors 5-fluoro-2-indolyl des-chlorohalopemide hydrochloride hydrate (FIPI), sodium metavanadate (NaVO_3_), and sodium tungstate hydrate (Na_2_WO_4_) were obtained from Sigma–Aldrich Sweden AB (Stockholm, Sweden).

The DBS filter paper cards and devices for microsampling of blood examined were the Whatman 903 Protein Saver card (GE Healthcare Ltd., Cardiff, UK), the Capitainer quantitative DBS (qDBS; Capitainer AB, Stockholm, Sweden), the 10 μL Mitra Clamshell (Neoteryx, Torrance, CA, USA), and the HemaXis DB 10 (DBS System SA, Gland, Switzerland). The addition of PLD inhibitors to dismounted Capitainer qDBS discs was made by pipetting 2-μL inhibitor solution onto the disc and allowing it to dry for at least 24 h.

### Blood samples

The blood specimens used for this study were de-identified surplus volumes of fresh venous whole blood samples selected among those sent to the Departments of Clinical Pharmacology and Clinical Chemistry, Karolinska University Laboratory (Stockholm) for routine analysis. The blood was collected in EDTA tubes and stored at 4 °C where PEth is reported to be stable for at least 3 weeks [4, 10]. The possible presence of ethanol was not tested for.

The blood was spiked with ethanol to a final concentration of 2 g/L, mixed, and immediately used for the experiments (i.e., applied on the filter paper cards or microsampling devices), or stored at room temperature (~20–22 °C). Before use, the microsampling devices were left to dry at room temperature for at least 3 h, unless otherwise stated.

The procedures followed were approved by the ethics committee at the Karolinska University Hospital (No. 2013/341-31/4).

### LC–MS/MS measurement of PEth 16:0/18:1

Measurement of PEth 16:0/18:1 was done by LC–MS/MS at SYNLAB Medilab (Stockholm, Sweden), essentially as previously described for liquid whole blood, DBS samples, and other biological matrices [11, 27, 32, 33]. The instrument was a Sciex Qtrap 5500 LC–MS/MS system (AB Sciex LP, Ontario, Canada), and the analytical column was a Kinetex 2.6 μm XB-C18 100 Å, 30 × 2.1 mm (Phenomenex Inc., Torrance, CA, USA). The extraction of PEth from the dried blood microsampling devices was done as previously described, except that the extraction solvent was the Extraction buffer 1 from the PEth LC–MS/MS kit (RedHot Diagnostics AB, Södertälje, Sweden).

The lower limit of quantification of PEth in liquid blood was 0.01 μmol/L. The lowest quality control sample (~0.10 μmol/L) had an analytical imprecision (CV) of 7.0% and the accuracy of the method was ascertained by participation in a proficiency testing program for PEth in liquid blood (Equalis AB, Uppsala, Sweden). The CV for PEth measurement in blood collected on intact qDBS devices was determined to be 15.2% (*N* = 10) at 0.02 μmol/L. The CV for the other devices was not determined.

## Results

### Initial experiments with the Capitainer qDBS device

**Fig. 1:**
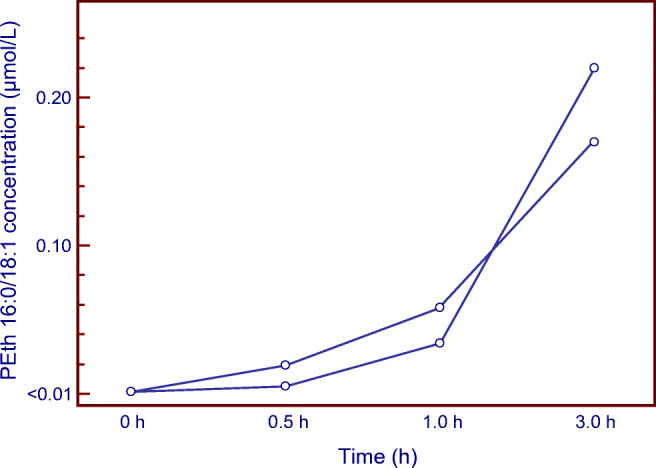
Formation of phosphatidylethanol (PEth 16:0/18:1) in two human blank whole blood samples fortified with 2 g/L ethanol during drying on a Capitainer qDBS microsampling device at room temperature

Despite previous reports that collecting blood on filter paper eliminates the risk for post-sampling formation of PEth [28, 30, 34], it was observed during validation of the Capitainer qDBS device that PEth was formed from ethanol during drying. When whole blood samples from two donors, both testing negative for PEth (i.e., < 0.01 μmol/L), were fortified with 2 g/L ethanol before being applied onto the qDBS devices, a continuous formation of PEth was observed during drying, with concentrations of 0.22 and 0.17 μmol/L, respectively, after storage for 3 h at room temperature (Fig. 1).

To further investigate this observation, filter discs dismounted from the Capitainer qDBS devices were fortified with 0.20 μg of the PLD inhibitor FIPI [23], before applying 10 μL of ethanol-spiked PEth-negative blood (i.e., the same volume as collected with the intact devices). Dismounted discs without addition of FIPI served as controls. After leaving the discs to dry for 3 h at room temperature, formation of PEth occurred in the control discs not containing FIPI, albeit at lower levels (0.03 and 0.04 μmol/L, respectively) than with the intact qDBS devices, whereas no PEth formation was observed (< 0.01 μmol/L) in the discs fortified with FIPI.

### Investigation of alternative PLD inhibitors

Two inorganic chemicals known to decrease PLD activity, NaVO_3_ and Na_2_WO_4_ [35], were evaluated as alternative inhibitors of post-sampling PEth formation. In a first experiment, filter discs dismounted from Capitainer qDBS devices were fortified with 25 μg NaVO_3_ or 65 μg Na_2_WO_4_ (0.20 μmol), each in 2 μL, before the addition of PEth-negative blood spiked with 2 g/L ethanol. No PEth formation (i.e., < 0.01 μmol/L) was observed during drying at room temperature with either substance. In a subsequent concentration–response experiment, NaVO_3_ was found to be the more potent PLD inhibitor (data not shown) and was therefore selected for further inhibition studies.

**Fig. 2:**
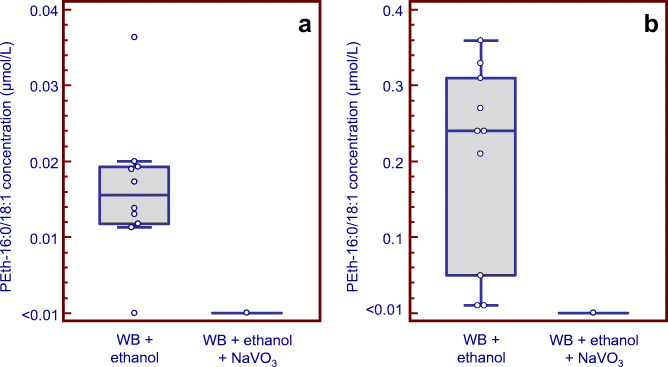
Box-and-whisker plots showing the formation of PEth 16:0/18:1 in **a** human blank whole blood (WB) samples from 10 individuals fortified with 2 g/L ethanol and applied and dried on dismounted Capitainer qDBS discs, with or without addition of NaVO_3_ (25 μg/disc), and **b** blank whole blood samples from 10 other individuals fortified with 2 g/L ethanol and added on standard qDBS microsampling devices or devices fortified with 25 μg NaVO_3_ per filter disc. The drying time was 3 h at room temperature. The PEth results in the presence of PLD inhibitor were always below the lower quantification limit (< 0.01 μmol/L). Please note the difference in scale in panels **a** and **b**

The ability of NaVO_3_ (25 μg/dismounted disc) to block post-sampling formation of PEth in blood spiked with 2 g/L ethanol was confirmed, in a separate experiment using blood specimens from 10 different individuals (Fig. 2a). Furthermore, when ethanol-spiked blood from 10 other individuals was applied to intact prototype devices of the Capitainer qDBS fortified with 25 μg NaVO_3_ per filter disc and examined after storage at room temperature for 3 h, no formation of PEth was observed. In contrast, with the standard qDBS discs without inhibitor, PEth formation was demonstrated in 8 of 10 blood samples, albeit at highly variable rate (range 0.01–0.36, median 0.24 μmol/L PEth) (Fig. 2b).

To confirm that post-sampling formation of PEth occurred also in PEth-positive blood, the latter experiment was repeated with four blood samples containing 0.48–1.1 μmol/L PEth. After spinking the blood samples with 2 g/L ethanol, no further formation of PEth was observed in the qDBS devices fortified with NaVO_3_ (mean 101%, range 99–105%, of the starting value)_,_ whereas post-sampling formation occurred (mean 142%, range 130–165%, of the starting value) in the original devices without PLD inhibitor.

### Study of PEth formation in commercial dried blood microsampling devices

**Fig. 3:**
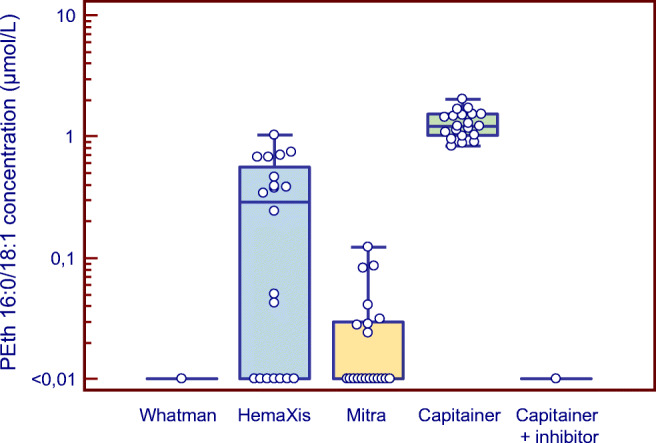
Box-and-whisker plots showing the formation of PEth 16:0/18:1 in human blank whole blood samples from 20 individuals fortified with 2 g/L ethanol and applied on filter paper (Whatman 903 Protein Saver card) or on three commercial devices for dried blood microsampling (HemaXis DB 10, 10 μL Mitra Clamshell, standard Capitainer qDBS, and qDBS devices fortified with 25 μg NaVO_3_ per filter disc). The storage time was 48 h at room temperature. The highest PEth concentrations were formed in the qDBS device (range 0.83–2.02, median 1.2 μmol/L; *N* = 20 samples), followed by HemaXis (0.04–1.04, median 0.39 μmol/L; *N* = 13), and Mitra (0.02–0.12, median 0.06 μmol/L; *N* = 8). No PEth formation was observed (< 0.01 μmol/L) with the Protein Saver card, or with the qDBS device fortified with NaVO_3_. When the ethanol-spiked blood samples were left in the test tubes for 48 h at room temperature, PEth was formed in all of them (range 0.03–0.08, median 0.04 μmol/L PEth)

Three commercial dried blood microsampling devices for collecting finger-pricked blood were examined for the risk of post-sampling formation of PEth from ethanol during drying. Blank blood from 20 individuals spiked with 2 g/L ethanol was applied onto the devices and let to dry for 48 h at room temperature. Formation of PEth during drying was noted in 8 of the 20 samples with the Mitra (range 0.02–0.12, median 0.04 μmol/L PEth), in 13 samples with the HemaXis (range 0.04–1.04, median 0.39 μmol/L PEth), and in all 20, and at highest levels, with the standard Capitainer qDBS (range 0.83–2.02, median 1.22 μmol/L PEth) devices. However, no formation of PEth was observed with the Whatman 903 Protein saver card or the Capitainer qDBS device fortified with the PLD inhibitor NaVO_3_ (Fig. 3).

For comparison, when the 20 blood samples spiked with ethanol were left in the test tubes for 48 h at room temperature, PEth formation was observed in all of them (range 0.03–0.08, median 0.04 μmol/L PEth).

## Discussion

Although PEth measurement in whole blood is considered a specific and sensitive alcohol biomarker that has become increasingly used, there are some important limitations to be aware of. At group level, the PEth value correlates fairly well with past weeks alcohol intake, and dose-response cutoffs to facilitate the interpretation of test results have been proposed [16, 17, 30]. Nevertheless, the large inter-individual scatter in the alcohol dose versus PEth response [18, 19, 36], and in the half-life after alcohol withdrawal [20], allows only an approximate estimate of the amount and time of previous drinking, and implies risk for misclassification between, for example, moderate and heavy drinking.

Another, and legally more important, concern relates to the risk for post-sampling formation of PEth in blood specimens containing ethanol [4, 12, 21, 37], which was further confirmed by the present results. PEth has both clinical and forensic applications as an alcohol biomarker [17, 38] and an increase in the concentration due to post-sampling formation may lead to an erroneous interpretation and cause unjustified negative consequences for the individual, which is not acceptable. It has been reported that a considerable proportion (12%) of specimens submitted for routine analysis of PEth contains ethanol [38], and as this subgroup of samples also showed the highest PEth concentrations, it calls for concern regarding a possible contribution from post-sampling formation. Although the presence of ethanol in a blood sample typically results from recent drinking [39], artifactual ethanol formation after sampling due to microbial action is a well-known problem in forensic toxicology [40]. Accordingly, finding ways to eliminate this risk is important [23].

Collecting finger-pricked blood on filter paper (DBS) instead of traditional venous blood sampling in vacutainer tubes has been reported to eliminate post-sampling formation of PEth [28, 30, 34], possibly due to the evaporation of ethanol during drying. This was further supported by the results of the present study, as no PEth formation was observed when blood spiked with ethanol at a physiologically relevant concentration (2 g/L) was added to Protein Saver card filters. However, when similar experiments were performed with three commercial devices for volumetric dried blood microsampling, which is considered important for use in test applications needing precise substance quantification [41, 42], post-sampling formation of PEth from ethanol was observed with all of them, albeit to varying degrees, upon drying and storage at room temperature. A likely cause for the difference is that the drying of the blood and the evaporation of ethanol occurred much slower in the devices, compared with on filter paper cards where drying takes place openly. This was further supported by the observation of smaller post-sampling formation of PEth in dismounted Capitainer qDBS filters compared with in the intact devices. It should be noted that post-sampling formation of PEth in the microsampling devices was generally higher compared with in the blood stored in test tubes. However, this observation might be influenced by a simultaneous risk for some PEth degradation in liquid blood stored at room temperature [22].

Using inhibitors of PLD, the enzyme responsible for PEth formation from ethanol and phosphatidylcholine, such as FIPI is another way to avoid post-sampling formation of PEth [23]. The present study demonstrated two inorganic salts of vanadate and tungstate (NaVO_3_ and Na_2_WO_4_) as less expensive alternatives to the pharmaceutical FIPI as inhibitors of PLD. Both substances are phosphate mimetics that can interfere with the substrate binding and catalytic activity of enzymes in the PLD superfamily [35]. NaVO_3_ was selected for further use in this study, due to a higher potency. Accordingly, when evaluating a prototype of the Capitainer qDBS microsampling device where the filters had been fortified with NaVO_3_, no post-sampling formation of PEth was observed.

## Conclusion

The results of the present study confirmed previous observations that PEth can be formed in whole blood samples after collection, if they contain ethanol. This represents a major drawback when PEth is used as an alcohol biomarker, because it has clinical and forensic applications and a positive test result can have serious consequences. The results further confirmed that sampling and storing blood on standard filter paper (DBS) seemingly eliminated this risk, whereas post-sampling formation of PEth from ethanol occurred with all three commercial devices for volumetric dried blood microsampling. It is therefore recommended to use an inhibitor of PLD, for example, NaVO_3_, whether venous blood is collected in a vacutainer tube or finger-pricked capillary blood using devices for microsampling; otherwise, a PEth value can be questioned and disputed. If venous blood is used, ensuring there is no ethanol present is also an option.
